# An updated animal model capturing both the cognitive and emotional features of post-traumatic stress disorder (PTSD)

**DOI:** 10.3389/fnbeh.2014.00142

**Published:** 2014-04-29

**Authors:** Andrea Berardi, Viviana Trezza, Maura Palmery, Luigia Trabace, Vincenzo Cuomo, Patrizia Campolongo

**Affiliations:** ^1^Department of Physiology and Pharmacology, Sapienza University of RomeRome, Italy; ^2^Department of Science, University of Roma TreRome, Italy; ^3^Department of Clinical and Experimental Medicine, University of FoggiaFoggia, Italy; ^4^Sapienza School of Advanced Studies, Sapienza University of Rome Rome, Italy

**Keywords:** memory, predator odor, footshock, animal models of PTSD, social behavior, trauma, stress, rats

## Abstract

The new-released Diagnostic and Statistical Manual of Mental Disorders (DSM-5) defines post-traumatic stress disorder (PTSD) as a “trauma and stressor-related disorder”. PTSD pathogenesis relies on paradoxical changes of emotional memory processing induced by the trauma exposure and associated with emotional dysfunction. Several animal models of PTSD have been validated and are currently used. Each one mimics a particular subset of the disorder with particular emphasis, mainly driven by the past classification of PTSD in the DSM-4, on the emotional features. In view of the recent update in the DSM-5, our aim was to develop, by using well-validated paradigms, a modified model of PTSD able to mimic at the same time both the cognitive and emotional features of the disease. We exposed male rats to either a piece of worn cat collar or to a series of inescapable footshocks paired with a PTSD risk factor, i.e., social isolation. Animals were subsequently re-exposed to the conditioned contexts at different time intervals in order to test memory retention for the stressors. In addition, footshock-exposed rats were tested in the elevated-plus-maze and social interaction tests. We found that rats exposed to a cat collar exhibited an acute fear response that did not lead to enduring memory retention. Conversely, footshock-exposed rats expressed a successful retention of the stressful experience at 1, 7, 14, 21 and 56 post-exposure days. Footshock-exposed rats displayed an anxious behavioral profile in the social interaction test and a significantly reduced locomotor activity in the elevated-plus-maze test. These dysfunctions were not observed when animals were socially housed, thus highlighting a social buffering effect in the development of the pathology. Our results underline the good validity of a footshock-based paradigm paired with social isolation as a PTSD animal model, able to mimic at the same time both some of the enduring cognitive and emotional facets of the pathology.

## Introduction

Post-Traumatic Stress Disorder (PTSD) is a chronic psychiatric disorder triggered by a traumatic and/or life threatening event. Even if the majority of people experience at least one traumatic event during lifetime, only a subset ultimately develops PTSD (Breslau, 2009). According to the last edition of The Diagnostic and Statistical Manual of Mental Disorders (DSM-5), PTSD is a “trauma and stressor-related disorder” identified by eight diagnostic criteria (American Psychiatric Association, 2013): (a) stressor, i.e., the exposure to an intense source of stress; (b) intrusion symptoms (i.e., re-experiencing the trauma); (c) avoidance of trauma-related stimuli; (d) negative alterations in cognitions and mood; (e) alterations in arousal and reactivity; (f) duration of symptoms for at least 1 month; (g) functional significance (i.e., distress or functional impairments in different domains such as social or occupational); and (h) exclusion (of other possible causes for the symptomatology).

Consensus exists that dysregulation of emotional memory processes is a primary etiopathological factor for PTSD onset (Layton and Krikorian, 2002; Siegmund and Wotjak, 2006; de Quervain et al., 2009; Daskalakis et al., 2013; Parsons and Ressler, 2013). Many are the aberrant memory processes participating to PTSD development. Processes of over consolidation could take place right after any re-experiencing symptom, updating the traumatic memory and prolonging its persistence over time (de Quervain et al., 2009), thus leading to a failure of the extinction processes. This could ultimately account for the patient’s inability to update the aversive nature of trauma-related reminders into a “no more harmful” representation (Charney et al., 1993; Milad et al., 2009).

Studying the neural mechanisms involved in the development of PTSD in humans would require prospective studies. Therefore, animal models are of crucial importance to study the neural underpinnings of the pathology and to develop innovative treatments (Pitman et al., 2012).

The alterations in arousal, reactivity, mood and social functioning, accompanied by the cognitive dysfunction, make PTSD a highly complex pathology not easy to model in preclinical research. Although there is no animal model that can capture, at the same time, all the molecular, cellular and behavioral features of the disorder, the development of animal models able to mimic some of the features of the pathology is of great help (Berardi et al., 2012; Daskalakis et al., 2013; Trezza and Campolongo, 2013). In recent years, many rodent models of PTSD have been described (see Berardi et al., 2012; Cohen et al., 2012a; Daskalakis et al., 2013; Goswami et al., 2013; for comprehensive review). All the models are based on the exposure to an acute stressor. Among the stressors frequently used, the exposure to predator threat (Adamec et al., 2004; Zoladz et al., 2008, 2013) or predator odor (Zohar et al., 2008; Mackenzie et al., 2010; Cohen et al., 2012b) has received considerable interest. Inescapable electric shocks represent another frequently used source of stress in PTSD studies (Yamamoto et al., 2009). In addition, the Single Prolonged Stress (SPS) model involves the combined exposure to multiple stressors (i.e., restraint stress, forced swim and exposure to ether) (Liberzon et al., 1997; Yamamoto et al., 2009; Ganon-Elazar and Akirav, 2012; Knox et al., 2012; Eagle et al., 2013). Although these models have good degrees of face and construct validity, they present some limitations with regards to the new additions present in the DSM-5. The use of predator stress is mainly limited by the difficulty in modulating the intensity of the evoked response (Siegmund and Wotjak, 2006). Moreover, the evidence of different behavioral outcomes caused by different stimuli, e.g., different cats (Muñoz-Abellán et al., 2010) or synthetic vs. natural odors (McGregor et al., 2002; Staples and McGregor, 2006; Staples et al., 2008; Hacquemand et al., 2013) are compelling and limits the possibility of standardized and replicable results. On the other hand, the footshock-based models (e.g., contextual fear conditioning paradigms), when not paired to any risk factor for PTSD development (Pitman et al., 1993), only furnish a measure of a physiological cognitive response in terms of memory retention of the emotional event (Siegmund and Wotjak, 2006). The SPS model has the limit to use not a unique source of stress but rather several stressors, thus not perfectly mimicking the common set of trauma experienced by PTSD patients (Yamamoto et al., 2009). Among these stressors, the loss of consciousness, obtained by means of different anesthetic agents (e.g., ether, isoflourane), presents a two-fold problem: (i) anesthetics are known *per se* to differentially influence cognitive processes with the effects depending on the specific drug, the dose, the type of memory, the experimental paradigm, the species and age of the experimental subject, thus making it difficult to have replicable results (Hauer et al., 2011; Hemmings and Mackie, 2011; Wang and Orser, 2011; Berardi et al., 2012; Goswami et al., 2013); and (ii) the use of anesthetics becomes an important confounding factor when evaluating potential new drugs, because of the large possibility of drug-drug interactions impossible to control.

The dissection of the mnestic content of the disease from its emotional consequence may pave the way to the discovery of pharmacological tools acting not only on PTSD symptoms but also on its causes (i.e., cognitive dysfunction). In this context, the ability of a PTSD animal model to evaluate at the same time both the cognitive and emotional aspects of the disease, by means of a combination of different already validated paradigms, has been proposed as a particularly attractive perspective (Siegmund and Wotjak, 2006; Berardi et al., 2012). Hence the objective of the present work was to develop an animal model useful to mimic and consequently study some of cognitive and emotional dysfunctions of PTSD at the same time in the same animal. To this aim we exposed male rats to a source of acute stress. In a first series of experiments we exposed animals to a cat collar. In a second set of experiments we exposed rats to a single exposure of multiple brief inescapable footshocks and we paired this stressor with a risk factor for PTSD development such as social isolation. More importantly, since PTSD is a chronic and persistent disorder, our second goal was to induce in the stress-exposed animals a form of measurable fear memory and emotional dysfunction persisting at very long retention intervals extending far beyond the standard timings usually considered.

## Materials and methods

### Subjects

Adult male Sprague-Dawley rats (weighting 350–450 g and aging 2 months at the time of testing; Charles River Laboratories, Calco, Italy) were kept in an air-conditioned controlled colony room (temperature: 21° ± 1°C; lights on from 7:00 a.m to 7:00 p.m.) with food and water available *ab libitum*. All the experiments were run during the light phase of the cycle. Rats were handled for 1 min each once a day for 7 consecutive days before behavioral testing. All procedures were performed in compliance with guidelines from the Italian Ministry of Health (law D.L. 116/92) and the European Communities Council Directive (2010/63/EU).

### Behavioral procedures

**Figure 1:**
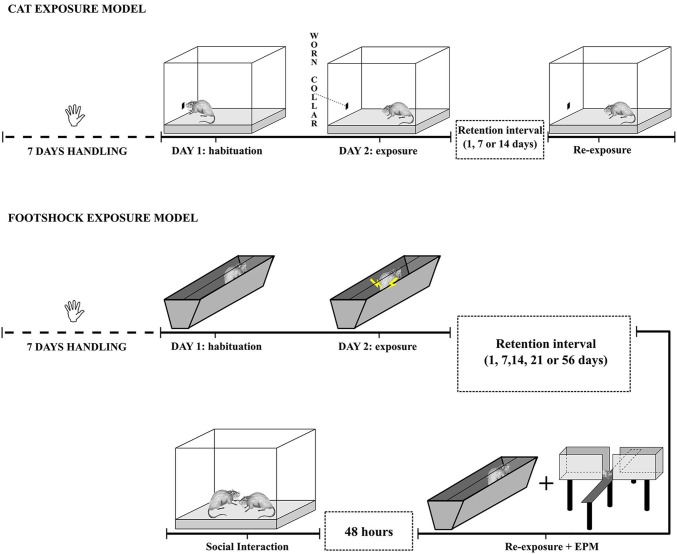
**Schematic representation of the cat odor and footshock exposure PTSD models**.

All the experimental sessions were video-recorded and subsequently scored by two experts and well trained researchers blind to the experimental conditions. For a graphical representation of the two models, see Figure 1.

#### Cat odor exposure model

All behavioral procedures took place in a sound-attenuated room with dim illumination (~ 10 lux inside the test arena). The test arena consisted in a quadrangular box (40 × 40 × 60 cm, l × w × h) made of transparent Plexiglas. The floor was covered with 4 cm of clean sawdust. After each session, fecal boli were removed, sawdust was blended, and the arena’s walls were cleaned with a 70% pure ethanol solution.

##### Housing

Rats were housed in groups of three per cage and were isolated from 3 days prior the habituation session until the end of the behavioral testing.

##### Habituation

Rats were individually taken from the home-cage and habituated for 20 min to the test arena in order to reduce novelty-induced stress. A small piece (2.5 cm) of unworn neoprene cat collar was attached with paper tape at the center of one of the arena’s walls, approximately 4 cm above the sawdust level. At the end of the 20 min rats were returned to their home cage. The choice of the aversive stimuli was based on previous works (Dielenberg et al., 2001).

##### Exposure session

The day after, half of the rats were randomly assigned to the exposed group (EXP) while the other half were assigned to the unexposed control group (UNEXP). UNEXP rats received another 20 min session in the arena with an unworn cat collar, as in the habituation session. EXP rats received a 20 min session in the arena but with a piece of cat collar that was worn by a cat for 30 consecutive days. Worn cat collars were kept in polyethylene zipped bags at −20°C and put at room temperature 2 h prior the exposure session. To avoid any possible contamination of cat odor to UNEXP rats, different but identical arenas for the two conditions were used. Moreover, experimenters always changed their gloves after the placement of the worn collar into the arena.

##### Re-Exposure session

One, 7 or 14 days after the exposure rats from both EXP or UNEXP groups received another 20 min session in the arena with an unworn collar in it, as in the habituation session, in order to be tested for successful memory retention of the stressful experience.

Behavioral measures taken into account during both the exposure and the re-exposure sessions were: (i) percentage of freezing time; and (ii) crossing, wall rearing and rearing frequencies. Freezing was defined as the complete lack of movement except for those necessary for respiration (Fanselow, 1982) and was measured during the re-exposure session as a measure of memory retention. For crossing measurement, a grid drawn on a transparent sheet and dividing the arena into 16 identical sectors was superimposed to the monitor during data collection, and a single crossing was defined as the rat passing from a square to another with all the four paws. Rearing was defined as the rat standing on the hind legs and wall rearing was defined as the rat standing on the hind legs and with the forepaws touching the inner side of the arena’s walls.

#### Footshock exposure model

The footshock exposure procedure was conducted in a metal trough-shaped box 60 cm long, 15 cm deep, 20 cm wide at the top and 6.4 cm wide at the bottom made of two metal plates connected to an animal shocker. The apparatus was placed into a dimly illuminated and sound-attenuated room. After each session, fecal boli were removed and the apparatus was cleaned with a 70% pure ethanol solution. Illumination was provided by a 25W white light bulb to one corner of the room (~0.40 lux inside the apparatus).

##### Housing

To evaluate whether social isolation could be a detrimental factor in the development of PTSD symptoms, rats were housed in two different conditions (isolation or social housing) and different cohorts of both conditions were tested in each experiment. Rats from the isolation condition were isolated 3 days prior the habituation session until the end of the behavioral testing. Rats from the social housing condition were always housed in groups of three and were isolated 24 h before the social interaction test in order to increase their motivation to interact.

##### Habituation

On the first day of testing, rats were individually taken from the home-cage and habituated for 5 min to the test apparatus. At the end of the 5 min, rats were returned to their home cage.

##### Exposure session

The day after, rats were divided in two different groups: exposed (EXP) and unexposed control group (UNEXP). The footshock exposure procedure consisted in a slightly modified version of the one used by Chen et al. (2012). EXP rats were individually placed in the apparatus and were left undisturbed for 2 min. After that, five footshocks (2 s, 0.8 mA) were randomly delivered with the last always given at the end of the fifth minute. Inter-shock intervals were randomized by a scrambler and were used in order to avoid any form of temporal conditioning. After the last shock, rats were kept in the apparatus for 60 additional seconds to facilitate the context association to the aversive stimuli. UNEXP rats received the same behavioral procedure except that no shock was delivered.

##### Re-Exposure session

Separate cohorts of rats of both the EXP and UNEXP groups were re-exposed to the apparatus 1, 7, 14, 21 or 56 days after the exposure session for the isolation condition, and 1, 7 and 14 days after the exposure session for the social housing condition (longer time points for the social housing conditions were not taken into consideration since no alterations were appreciated 14 days after the exposure). Memory retention was evaluated over a 10-min period by analyzing contextual freezing behavior (Chen et al., 2012).

To evaluate the level of emotional distress, both the EXP and UNEXP rats were tested in two well validated animal models used to assess emotionality in rats: the elevated plus maze (EPM) test, immediately after the re-exposure session, and the social interaction (SI) test 48 h after the re-exposure session.

### Elevated plus maze test

The EPM was performed following the procedure used by Bortolato et al. (2006) and Trezza et al. (2008). The EPM comprised a central platform 10 × 10 cm, two opposed open arms 50 × 10 cm and two opposed closed arms 50 × 10 × 40 cm. The floor of the maze was made of black Plexiglas while the walls were made of transparent Plexiglas and was elevated 60 cm above the floor level. The EPM was conducted under red light illumination (~5.5 lux on the apparatus). Immediately after the 10 min re-exposure session each rat was taken from the apparatus and placed in the central platform of an EPM facing one of the enclosed arms. The EPM session lasted 5 min after which the rat was returned to the home cage. After each session, fecal boli were removed, and the maze was cleaned with a 70% pure ethanol solution. The measured behavioral parameters were the percent of time spent in the open arms, the percentage of entries in the open arms, the number of entries in the closed arms, the number of stretched attend postures (SAP), i.e., when the rat stretches forward and retracts back afterward, and the number of head dips.

### Social interaction test

The SI test was performed following the procedure used by Segatto et al. (2014). Couples for the SI test were decided according to the following criteria: (1) belonging to the same experimental condition; (2) unfamiliarity, i.e., the two rats of each pair were not housed in the same cage; and (3) least weight difference. Each couple was put for 10 min in a quadrangular arena (40 × 40 × 60 cm; l × w × h) made of transparent Plexiglas with 4 cm of clean sawdust covering the floor, under red lights conditions (~10 lux). After each session, rats were returned to their home-cage, fecal boli were removed, sawdust was blended, and the arena’s walls were cleaned with a 70% pure ethanol solution. In this test, non-social behaviors i.e., wall rearing, rearing and crossing behaviors were scored as previously described and social behaviors were described as follows. Following was defined as one rats following the direction of the other, sniffing was defined as one rat sniffing the other in any part of the body, pouncing was defined as one rat nosing or rubbing the nape of the neck of the other, pinning was defined as the rotation of one rat to its dorsal surface after receiving a pounce from the other, boxing was defined as both rats standing on the hind legs in front of each other moving the forepaws, crawling over was defined as one rat passing over the back of the other. The social interaction time was obtained by summing together all the discrete durations of each social behavior.

### Statistical analysis

Statistical analysis was performed using the SPSS statistical software. Each measure is expressed as mean ± SEM. For each behavioral measure Student’s *t*-test between UNEXP and EXP groups was performed in cat exposure sessions. In all the other cases, two-way ANOVAs with condition and time interval as between-subjects factors were used and Tukey-Kramer *post-hoc* test was performed to control for significant differences between groups. Significance was considered for *p* < 0.05.

## Results

### The cat collar exposure induces an acute fear response accompanied by a rapidly decaying contextual fear memory

**Figure 2:**
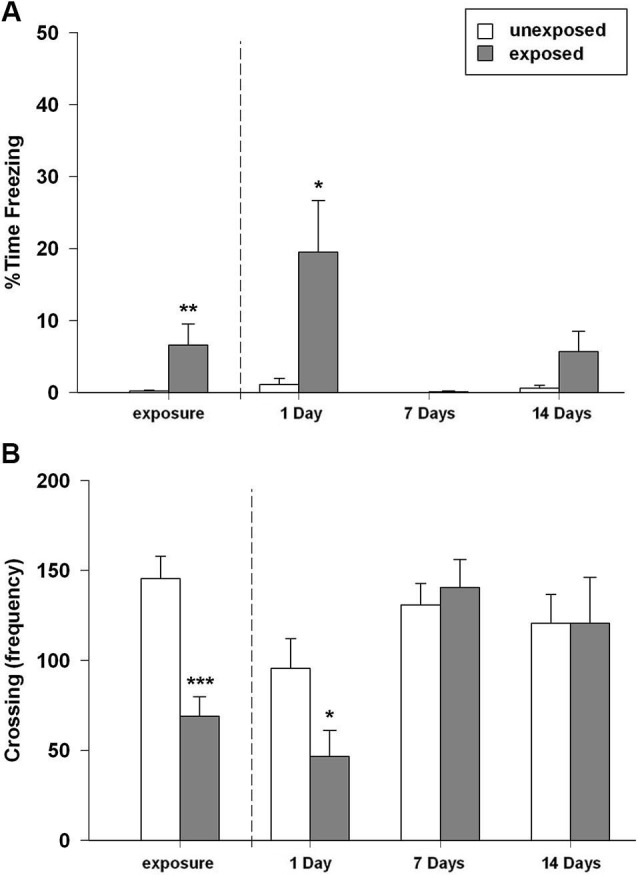
**(A)** Percentage of time spent in freezing and **(B)** number of crossings of rats exposed to an unworn (unexposed) or to a worn (exposed) cat collar during the exposure session (left plot) (EXP, UNEXP: *n* = 12) and re-exposure sessions performed 1, 7, or 14 days after the exposure to the stressor (right plots) (1D EXP *n* = 8, UNEXP *n* = 7; 7D EXP, UNEXP *n* = 9; 14D EXP *n* = 9, UNEXP *n* = 8). Data are expressed as mean + SEM (* *p* < 0.05; ** *p* < 0.01; *** *p* < 0.001; EXP vs. UNEXP).

In this experiment we aimed to investigate the rats’ memory retention for the stressful experience represented by the exposure to a worn cat collar at different time intervals. We found that rats exposed to worn cat collars displayed an acute intense and robust fear response during the exposure, as highlighted by longer freezing time in comparison to rats exposed to unworn piece of cat collars (*t*_22_ = −2.169, *P* = 0.041; Figure 2A). During the exposure session EXP rats, compared to UNEXP controls, showed a reduced motor activity in term of number of crossings (*t*_22_ = 4.669, *P* < 0.001; Figure 2B), rearings (*t*_22_ = 3.386, *P* = 0.003, mean EXP = 20.17 ± 3.65; mean UNEXP = 43.08 ± 5.70) and wall rearings (*t*_22_ = 4.010, *P* = 0.001, mean EXP = 30.50 ± 3.64; mean UNEXP = 57.83 ± 5.76). In the re-exposure sessions, the two-way ANOVA for freezing time with exposure condition and time intervals as between factors, revealed a significant main effect of exposure condition (*F*_1,44_ = 9.593, *P* = 0.003), a significant main effect of time intervals (*F*_2,44_ = 5.566, *P* = 0.007) and a significant condition × interval interaction (*F*_2,44_ = 4.450, *P* = 0.017). Two-way ANOVA for crossing, rearing and wall rearing frequencies showed no significant main effect of condition, a significant main effect of time intervals (*F*_2,44_ = 7.097, 8.515, 6.741; *P* = 0.002, 0.001, 0.003, respectively) and no significant condition × interval interaction. *Post-hoc* analysis indicated that rats re-exposed to the cat odor-paired context made significantly more freezing (*P* < 0.05; Figure 2A); less crossings (*P* < 0.05; Figure 2B), and less rearings (mean EXP = 13.25 ± 5.45; UNEXP = 26.00 ± 5.96, not shown in figure) when compared to their unexposed controls 1 day after the exposure. However, no such differences between EXP and UNEXP groups were detectable either 7 or 14 days after the exposure (freezing Figure 2A, crossings Figure 2B, rearings not shown in figure). The weak mnestic performance of exposed rats cannot be attributable to latent inhibition caused by prior habituation to the arena and collar stimuli. Indeed, the replication of the 7 days experiment without the habituation session, did not alter behavioral profile of exposed rats (data not shown). Taken together, these results demonstrated the inability of the predator stress model, at least in the experimental conditions used in the present work, to induce a long-lasting form of memory. Therefore, in accordance with the 3R principles of the European law for animal research, to reduce the number of animals used and their distress, rats socially housed were not tested and the emotional parameters were not evaluated.

### The exposure to multiple inescapable footshocks, paired with social isolation, induces a long-lasting contextual fear memory and emotional dysfunction. Social housing reduces the adverse consequences of trauma exposure on emotional dysfunction

In this experiment, we aimed to investigate memory retention for the stressful experience represented by the exposure to a series of five consequent footshocks. Freezing has been evaluated as a measure of memory retention.

**Figure 3:**
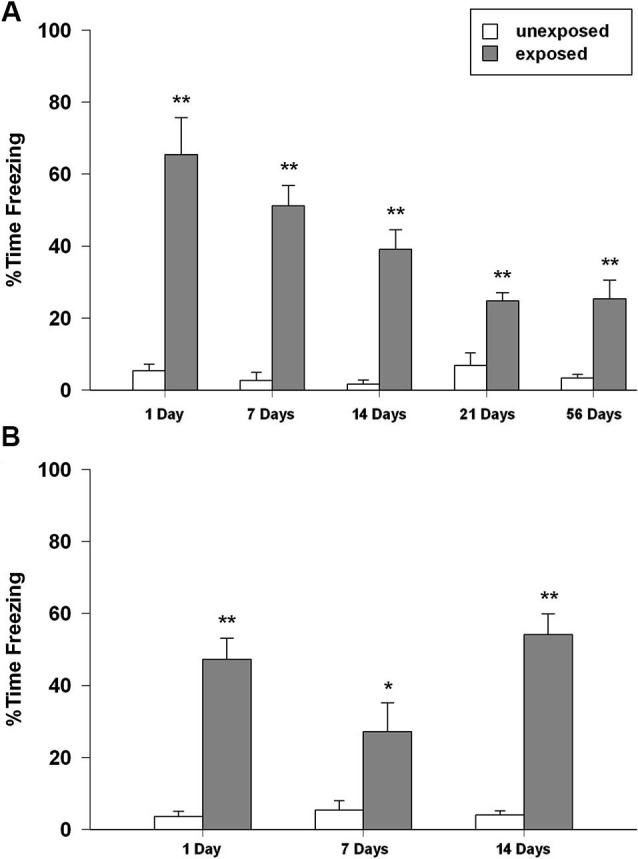
Percentage of time spent in freezing of rats exposed to the context and not shocked (unexposed) or exposed to the context and given five inescapable footshocks (exposed) during the re-exposure sessions performed 1, 7, 14, 21 and 54 days after the exposure (*n* = 8–11). **(A)** Isolated, **(B)** social-housed animals. Data are expressed as mean + SEM (* *p* < 0.05; ** *p* < 0.01; EXP vs. UNEXP).

In isolated animals, two-way ANOVA for freezing revealed a significant main effect of exposure condition (*F*_1,89_ = 158.548, *P* < 0.001), of re-exposure time intervals (*F*_4,89_ = 6.773, *P* < 0.001), and condition × intervals (*F*_4,86_ = 7.092, *P* < 0.001). *Post-hoc* analysis showed that EXP rats spent significantly longer time in freezing at any re-exposure interval than the UNEXP control group did (*P* < 0.01 for all the tested intervals; Figure 3A).

With regard to group-housed rats, ANOVA revealed a significant effect of exposure condition (*F*_1,47_ = 91.449, *P* < 0.001), time intervals (*F*_2,47_ = 3.493, *P* = 0.039), and condition × interval interaction (*F*_2,47_ = 4.475, *P* = 0.017). *Post-hoc* analysis revealed that EXP rats in the re-exposure session expressed significantly higher freezing rates than UNEXP animals (*P* < 0.01 for 1 and 14 days after exposure, *P* < 0.05 for 7 days after exposure; Figure 3B).

### Emotional behavior

We investigated the emotional profile of the footshock-exposed animals in the EPM and SI test, in order to assess whether the long-lasting memory retention was accompanied by any enduring change in emotional reactivity.

### Elevated plus maze

Results from the EPM failed to demonstrate a classical anxious profile in the EXP animals at all tested intervals and for both housing conditions. Interestingly, socially isolated EXP rats always made significantly less entries into the closed arms, an index of locomotory activity. Similar results were obtained in isolated rats that underwent EPM procedure 7 days after footshock exposure but without being re-exposed to the footshock-paired context (data not shown). This indicates that the EPM performance was not altered by prior re-exposure to the footshock-paired context.

#### Isolated rats

**Figure 4:**
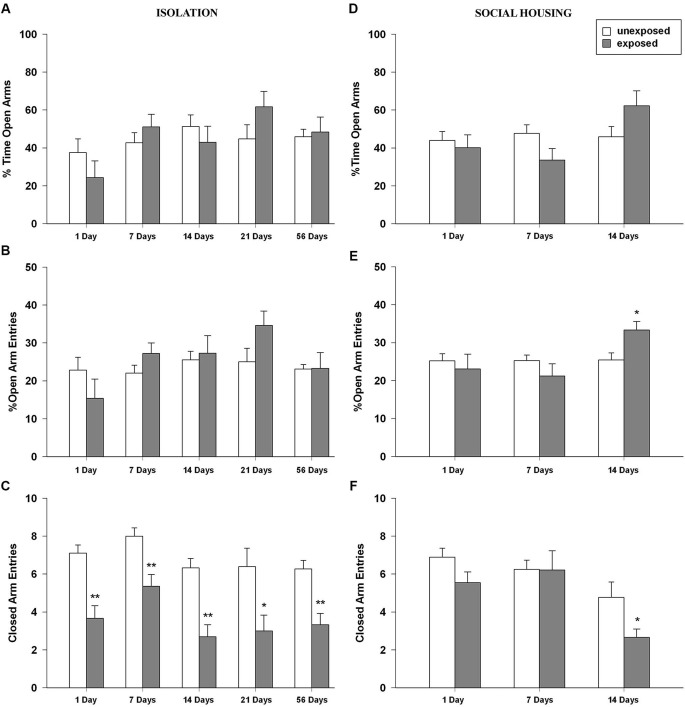
**(A,D)** Percentage of time in the open arms, **(B,E)** percentage of open arm entries and **(C,F)** closed arms entries of rats unexposed or exposed to the footshock in the EPM 1, 7, 14, 21 days after exposure to the stressor (*n* = 8–11). Data are expressed as mean + SEM (* *p* < 0.05; ** *p* < 0.01).

ANOVA for percent time in the open arms revealed no significant main effect of exposure condition, a significant main effect of time intervals (*F*_4,89_ = 2.775, *P* = 0.032) and no significant condition × interval interaction (Figure 4A). With regard to the percentage of entries in the open arms, ANOVA revealed no significant effects of exposure condition, a main effect of time intervals (*F*_4,89_ = 2.433, *P* = 0.053) and no significant condition × interval interaction (Figure 4B). Regarding the number of entries in the closed arms, significant main effects of exposure condition (*F*_1,89_ = 60.185, *P* < 0.001) and time intervals were found (*F*_4,89_ = 3.646, *P* = 0.008) but no significant condition × interval interaction was observed. Tukey *post-hoc* tests showed that EXP rats made significantly less entries in the closed arms of the maze compared to unexposed control rats for all the tested intervals (1, 7, 14, 54 post-exposure days: *P* < 0.01; 21 post-exposure days interval *P* < 0.05; Figure 4C). With regard to the number of head dips, ANOVA showed a significant main effect of exposure condition (*F*_1,89_ = 7.429, *P* = 0.008), of time time intervals (*F*_4,89_ = 4.615, *P* = 0.002) but no significant condition × interval interaction. The ANOVA for SAP frequency revealed a trend toward significance for exposure condition (*F*_1,89_ = 3.737, *P* = 0.056), a significant main effect of time intervals (*F*_4,89_ = 2.600, *P* = 0.041) and no significant condition × interval interaction.

#### Social-housed rats

One-way ANOVAs for the percent time spent in the open arms of the EPM revealed no significant main effect of exposure condition or time intervals but a significant condition × interval interaction (*F*_2,47_ = 3.229, *P* = 0.049). *Post-hoc* analysis did not reveal any difference between EXP and UNEXP rats (Figure 4D). In the percentage of open arm entries, the ANOVA revealed no significant effects of condition, a significant main effect of time intervals (*F*_2,47_ = 3.265, *P* = 0.047) and a trend toward significance for condition × interval interaction (*F*_2,47_ = 3.026, *P* = 0.058). Tukey *post-hoc* tests showed that at 14 post-exposure days, EXP rats made a higher percentage of entries in the open arms than unexposed controls (*P* < 0.05; Figure 4E). ANOVA for the number of closed arm entries showed a significant main effect of condition (*F*_1,47_ = 4.435, *P* = 0.041) a significant main effect of time intervals (*F*_2,47_ = 9.345, *P* < 0.001) and no significant condition × interval interaction. *Post-hoc* analysis showed that at 14 post-exposure days, EXP rats made significantly less entries in the closed arms of the EPM than unexposed control rats (*P* < 0.05; Figure 4F). For frequencies of head dips ANOVA showed no significant main effects of exposure condition, time intervals or condition × interval interaction. For numbers of SAPs ANOVA showed no significant main effect for exposure condition a significant main effect for time intervals (*F*_2,47_ = 11.889, *P* < 0.001) and no significant condition × interval interaction.

### Social interaction test

#### Isolated rats

Stress-exposed animals displayed an enduring alteration in social behavior.

**Figure 5:**
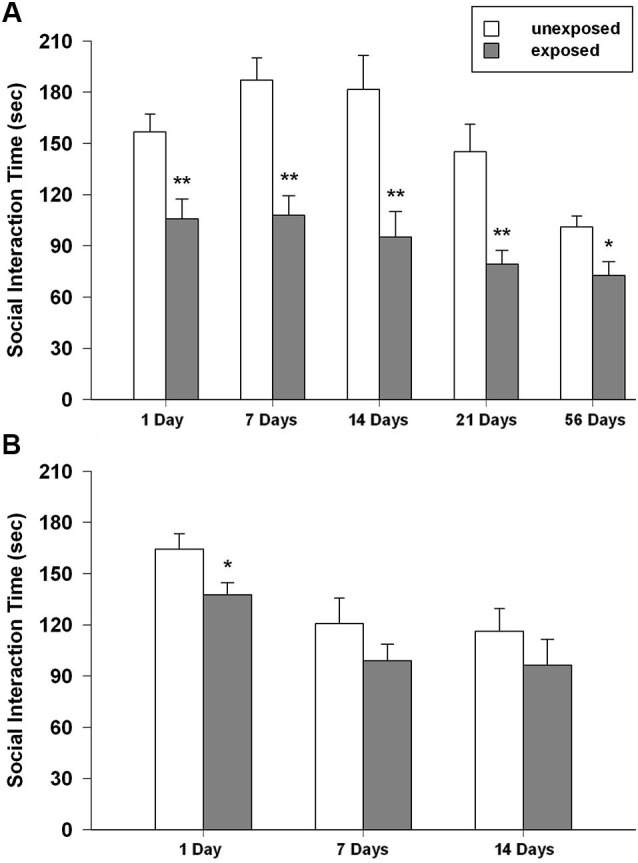
Time spent in social interaction of unexposed and exposed rats 1, 7, 14, 21 and 54 days the exposure to the stressor (*n* = 8–11). **(A)** Isolated, **(B)** social-housed animals. Data are expressed as mean + SEM (* *p* < 0.05; ** *p* < 0.01).

Rats previously exposed to the footshock spent less time interacting with the social partner than unexposed controls did. With regard to the total time of social interaction ANOVA revealed a significant main effect of exposure condition (*F*_1,89_ = 63.501, *P* < 0.001) and time intervals (*F*_4,89_ = 8.090, *P* < 0.001), but no significance for the condition × interval. *Post-hoc* comparisons showed that exposed animals interacted less time with the social partner than unexposed animals did at all the tested intervals (*P* < 0.01 from 1 to 21 post-exposure days; *P* < 0.05 for 54 post-exposure days; Figure 5A). The ANOVA for the number of crossings revealed no significant effect of exposure condition, a significant effect of time intervals (*F*_4,89_ = 5.098, *P* = 0.001) and a significant condition × interval interaction (*F*_4,89_ = 3.659, *P* = 0.008). Tukey *post-hoc* tests showed that rat re-exposed to the footshock-paired context 1 day after the exposure made significantly more crossings than the respective unexposed control group (*P* < 0.05; mean EXP = 421.89 ± 37.28; UNEXP = 321.56 ± 16.67; data not shown in figure).

#### Social-housed rats

In group-housed rats, the ANOVA for social interaction time showed a significant effect of exposure condition (*F*_1,47_ = 5.514, *P* = 0.023) and time intervals (*F*_2,47_ = 8.862, *P* = 0.001) but no significant condition × interval interaction. *Post-hoc* tests demonstrated that, EXP rats compared to UNEXP controls displayed less social interaction only 1 day after the exposure (*P* < 0.05, Figure 5B). The ANOVA for crossings showed a significant effect of exposure condition (*F*_1,47_ = 19.303, *P* < 0.001) and time intervals (*F*_2,47_ = 48.588, *P* < 0.001) but no significant condition × interval interaction was found. *Post-hoc* analysis revealed an augmented number of crossings for EXP rats compared to the UNEXP controls for all the tested interval (*P* < 0.01 for 1 post-exposure day, crossing mean EXP = 689.56 ± 25.26, UNEXP = 588.00 ± 11.46; *P* < 0.05 for 7 and 14 post-exposure days: 7 days crossing mean EXP = 423.44 ± 37.62, UNEXP = 306.25 ± 26.20, 14 days crossing mean EXP = 443.33 ± 49.78, UNEXP = 327.67 ± 18.99; data not shown in figure). Mean frequency scores ± SEM of the other behaviors from both the housing conditions are reported in the Table 1.

**Table 1:** **Mean frequencies of behavioral responses of the social interaction test**. (Data expressed as mean ± SEM).

**ISOLATION HOUSING CONDITION**									
		**Exploratory behaviors**		**Social behaviors**					
**Interval**	**Condition**	Wall Rearing	Rearing	Following	Sniffing	Pouncing	Pinning	Crawling over	Boxing
**1 DAY**	**UNEXP (*n* = 9)**	40.4 ± 3.4	27.7 ± 3.5	3.4 ± 0.8	51.2 ± 2.7	11.8 ± 2.7	2.9 ± 1.1	3.4 ± 1.1	0.9 ± 0.4
	**EXP (*n* = 9)**	42.9 ± 4.2	26.2 ± 3.4	5.2 ± 1.2	53.0 ± 5.0	4.8 ± 2.0	2.6 ± 1.7	4.1 ± 1.1	1.0 ± 0.6
**7 DAYS**	**UNEXP (*n* = 9)**	43.4 ± 5.3	24.9 ± 2.7	5.0 ± 1.3	79.7 ± 6.2	9.4 ± 2.0	4.7 ± 1.7	4.0 ± 1.7	2.1 ± 0.9
	**EXP (n = 11)**	45.2 ± 4.1	33.0 ± 3.0	2.5 ± 0.5	62.6 ± 5.9	4.5 ± 1.3	2.0 ± 0.7	2.9 ± 1.0	1.0 ± 0.4
**14 DAYS**	**UNEXP (*n* = 9)**	37.7 ± 3.1	29.3 ± 4.3	5.1 ± 1.3	64.0 ± 5.0	9.1 ± 1.4	5.2 ± 1.2	0.1 ± 0.1	2.8 ± 0.4
	**EXP (*n* = 10)**	38.0 ± 3.7	42.8 ± 5.0	2.8 ± 1.0	52.0 ± 6.0	2.8 ± 1.3	1.6 ± 0.9	1.5 ± 0.8	0.1 ± 0.1
**21 DAYS**	**UNEXP (*n* = 10)**	26.5 ± 3.0	21.2 ± 5.6	3.9 ± 1.6	54.8 ± 6.7	4.8 ± 1.4	2.4 ± 1.0	1.0 ± 0.5	6.6 ± 2.0
	**EXP (*n* = 10)**	30.7 ± 3.6	29.4 ± 2.5	2.3 ± 0.7	44.7 ± 4.1	1.9 ± 0.5	1.1 ± 0.3	0.8 ± 0.5	0.7 ± 0.3
**56 DAYS**	**UNEXP (*n* = 11)**	39.7 ± 4.1	38.6 ± 3.6	5.4 ± 1.0	66.1 ± 4.3	3.3 ± 1.0	1.8 ± 0.9	0.3 ± 0.2	2.1 ± 0.7
	**EXP (*n* = 11)**	37.4 ± 3.6	42.7 ± 3.2	2.0 ± 0.7	54.2 ± 4.5	2.6 ± 0.8	1.6 ± 0.6	0.4 ± 0.2	1.2 ± 0.3
**SOCIAL HOUSING CONDITION**									
**1 DAY**	**UNEXP (*n* = 9)**	50.1 ± 3.0	21.0 ± 4.5	6.7 ± 1.6	73.1 ± 6.1	13.2 ± 1.9	9.8 ± 1.6	1.7 ± 1.1	8.8 ± 2.0
	**EXP (*n* = 9)**	54.7 ± 4.0	25.4 ± 3.2	5.8 ± 1.0	63.1 ± 3.4	10.3 ± 1.4	9.3 ± 1.9	2.7 ± 0.8	2.9 ± 0.8
**7 DAYS**	**UNEXP (*n* = 8)**	47.0 ± 2.5	31.6 ± 6.5	4.0 ± 0.8	49.5 ± 3.1	5.9 ± 1.0	3.5 ± 1.0	0.4 ± 0.2	1.4 ± 0.6
	**EXP (*n* = 9)**	50.1 ± 5.7	37.8 ± 2.5	4.2 ± 1.2	54.7 ± 7.6	2.0 ± 0.8	1.3 ± 0.6	1.4 ± 0.8	1.0 ± 0.3
**14 DAYS**	**UNEXP (*n* = 9)**	44.6 ± 4.3	41.6 ± 4.0	4.7 ± 0.9	50.7 ± 5.7	7.6 ± 2.7	4.0 ± 2.1	1.4 ± 0.7	2.9 ± 0.8
	**EXP (*n* = 9)**	33.2 ± 2.4	39.8 ± 4.6	2.8 ± 0.8	45.7 ± 6.8	4.8 ± 2.6	3.6 ± 2.5	1.0 ± 0.4	2.6 ± 0.9

Data expressed as mean ± SEM.

## Discussion

The present findings show that: (a) exposure to cat collar, with the protocol used in the experiments described herein, is able to induce a short-lasting/rapidly decaying form of fear memory in rats; (b) the exposure to a series of inescapable footshocks, paired with social isolation, is able to induce in rats a long-lasting memory trace for the traumatic event, accompanied by enduring changes in social behavior; and (c) the social buffering operated by a social housing condition is able to importantly attenuate the emotional dysfunction observed in the footshock-exposed animals, while keeping the trauma-related memory unaltered. The phenotype obtained in the footshock-exposed isolated animals mimics some of the main behavioral PTSD symptoms recently listed in the DSM-5, i.e., long-term memory of trauma-related cues and sensitized behaviors such as social withdrawal.

### Cat odor exposure model

Exposure to a predator is among the methods most commonly used to mimic some features of PTSD; it has been demonstrated that it is capable to induce fear and anxiety as well as avoidance and defensive behaviors (Dielenberg and McGregor, 2001; Apfelbach et al., 2005; Takahashi et al., 2005). Fear-related behaviors are generally measured during a period of several minutes of exposure to the predator odor (Mackenzie et al., 2010; Cohen et al., 2012b). At the conclusion of testing, the animal is returned to its home-cage and tested the next day for retention of contextual fear behavior in the conditioning apparatus without further exposure to predator odor; freezing and reduced locomotion are considered as the main form of fear-related behavioral responses (Takahashi et al., 2008). Only few studies have measured fear and anxiety 7 days after the trauma exposure (Takahashi et al., 2005; Mackenzie et al., 2010; Cohen et al., 2012b) and rarely longer time points have been taken into consideration. Although at longer time points Mackenzie et al. (2010) were able to partially reproduce long-lasting conditioned alterations (e.g., locomotor activity), sensitized alterations (e.g., social withdrawal and deficit in the acoustic startle) were not detectable. In view of the above-mentioned evidence, criticism on the validity of the model has been raised mainly because of the difficulties to mimic enduring alterations (Muñoz-Abellán et al., 2009; Mackenzie et al., 2010). In particular, inconsistencies in the long-term effects of cat odor exposure have been frequently reported in the anxiety domain. For example, a dissociation in cat odor ability to induce conditioning without modifying anxiety has been reported (Muñoz-Abellán et al., 2009). In addition, dissociating effects of cat odor exposure on endocrine and behavioral parameters were also observed (Muñoz-Abellán et al., 2008). Therefore, in the present study we first tested whether the predator odor model was able to induce detectable long-term behavioral alterations both in term of conditioned and sensitized alterations. In accordance with literature evidence (Takahashi et al., 2005; Mackenzie et al., 2010; Cohen et al., 2012b), we found that rats re-exposed to the cat odor-paired context 24 h after the exposure session showed a robust freezing response and impaired locomotor activity, indicative of contextual fear. However, at longer retention intervals (i.e., 7 and 14 days after the exposure to the stressor) we did not appreciate any altered conditioned behavior indicative of augmented contextual fear memory. It has been argued that a valid animal model of PTSD should include a period of incubation after the stress exposure, after which the arising behavioral phenotype should persist unaltered or even worsen (Siegmund and Wotjak, 2006, 2007). Enduring alterations in fear conditioning, extinction learning, extinction retention and sensitization are involved in the development and/or maintenance of PTSD (Pitman et al., 2012). In this context, the predator odor undoubtedly remains a valuable model to test the behavioral dysfunction in the short-term. However, to appreciate long-term cognitive/emotional alterations different models are warranted.

### Footshock exposure model

With regard to other commonly used PTSD animal models, the footshock model does not reproduce a pathological phenotype only permitting to measure a physiological (and functionally relevant) form of emotional memory (i.e., the conditioned freezing response to a fearful context) (Pitman et al., 1993). To increase its face validity, we used a footshock model paired to one risk factors for PTSD, such as social isolation (Pitman et al., 1993). We found that, when the traumatic event consisted of a brief session of multiple footshocks, isolated animals displayed a contextual fear memory able to persist up to 8 weeks after exposure. This reveals the high stability of such learned response which may need a retention interval longer than 8 weeks or even of a lifetime to completely decay. To assess the presence and duration of both conditioned and sensitized behaviors, we additionally evaluated the emotional phenotype of the footshock-exposed rats in the EPM and SI tests. The EPM is a widely used paradigm for anxiety assessment and it is based on spontaneous rodent behavior involving the conflict between the exploration of a novel environment and its aversive characteristics (Pellow et al., 1985; File et al., 2004). Interestingly, in none of the tested intervals rats exposed to the footshocks showed a clear-cut anxious phenotype in this maze. Nevertheless, a significantly reduced locomotor activity (File et al., 2004) was systematically found in footshock-exposed rats when compared to unexposed animals, with exposed rats making less entries in the closed arms than controls did. This result is in line with literature data reporting hypoactivity in the EPM and other novel environments following contextual fear conditioning (Radulovic et al., 1998; Daviu et al., 2010). It is tentative to speculate that the footshock-induced trauma could sensitize the general responsiveness to stress thus reducing the rat activity in another stressful situation (i.e., EPM). This highlights the strong translational value in regard to the stress-sensitization occurring in PTSD patients (Siegmund and Wotjak, 2006; Brewin, 2011). An alternative specultaion for the reduction of entries in the closed arms of the EPM displayed by exposed rats could be represented by a generalization of fear; rats could systematically avoid the enlongated/narrow places with spatial configurations similar to the footshock-paired context. Noteworthy, the EPM may be not the ideal test to reveal the possible emotional dysfunction of footshock-exposed rats since the hypoactivity displayed by the exposed rats could mask a clear-cut anxiogenic-like profile. Therefore, the present results may help explaining the conflicting findings reported when the EPM is used as a measure of anxiety in PTSD animal models (Muñoz-Abellán et al., 2009). Conversely, here we show that the SI test (File et al., 2004) allows not only to evaluate the possible emotional alteration in traumatized animals but also to better mimic the human sensitized symptomatology. Animals exposed to the footshocks displayed reduced social interaction compared to unexposed control rats, thus showing augmented level of emotional distress during the social encounter or, with a different interpretation, less interest in social activities persisting up to 8 weeks after trauma exposure. These results are in line with previous studies reporting a reduction in social behavior in animals exposed to inescapable shocks (Maier and Minor, 1993; Short and Maier, 1993; Siegmund and Wotjak, 2007; Christianson et al., 2008).

### Effects of social housing on the footshock exposure model

To further examining the validity of our model we tested the hypothesis that lack of social support during the processing of the trauma might be an essential factor with respect to PTSD development. To this aim we subjected group-housed rats (social housing condition) to the above described footshock trauma model. Group-housed rats maintained a strong contextual fear memory at all the tested intervals as isolated animals did, but at the same time, they showed no enduring signs of emotional distress. In particular, the reduced time spent in social interaction by “traumatized” rats could only be detected 1 day after stressor exposure. This result could be attributed to a physiological emotional response to the acute stress and being not indicative of any pathological condition. If from one side it is tentative to speculate that the social support is able to reduce the adverse consequences of stress exposure and/or to enhance the ability to recover from stress, more appealing is the other side of the coin. The social isolation could be indeed considered a precipitating factor leading to an enhanced susceptibility to adverse emotional outcomes after trauma exposure. In both cases, our results are in line with previous findings showing long-term consequences of social defeat (and other stressors) on rodent behavior. Indeed, isolated rodents exposed to inescapable social defeat show long-lasting and adverse changes in behavior and physiology that are not observed, or are drastically reduced, in animals housed in groups (Ruis et al., 1999; Korte and de Boer, 2003; de Jong et al., 2005; Nakayasu and Ishii, 2008).

### Translational features of the footshock model

The reduced social interaction displayed by isolated animals is of high translational value with respect to the human diagnostic criteria of “feeling of being alienated from others” and “reduction in social functioning” which are two symptoms included respectively in the criterion D (i.e., negative alterations in cognitions and mood) and G (i.e., functional significance) of the DSM-5 (American Psychiatric Association, 2013). Moreover, the “markedly reduced interest in significant activities” (i.e., social interaction) and the “persistent inability to experience positive emotions” represent two more symptoms of the DSM-5 criterion D, which can be easily attributed to a blunted positive emotionality. Considering the highly rewarding value of social interaction in rats (see Trezza et al., 2011 for review), the reduced social interaction observed in footshock-exposed animals may be also indicative of a general blunting of positive emotions. Collectively, these results show that rats exposed to footshocks and housed in isolation express a strong contextual memory for the traumatic experience accompanied by social withdrawal, anxiety, and blunted emotionality symptoms. Importantly, all symptoms are persistent and do not undergo towards spontaneous recovery, underlining the chronic nature of the observed phenotype.

The possibility to mimic in the same animal both the cognitive (contextual) and emotional (sensitized) features of PTSD could open new research paths not only in term of a better understanding of the neural underpinning of the disorder but also for the testing of innovative drugs which could act at the same time by reducing the cognitive disability and ameliorating the emotional dysfunction observed in PTSD patients.

## Conflict of interest statement

The authors declare that the research was conducted in the absence of any commercial or financial relationships that could be construed as a potential conflict of interest.
